# Analytic solution for double optical metasurface beam scanners

**DOI:** 10.1038/s41598-022-09877-4

**Published:** 2022-04-08

**Authors:** Jingru Wang, Yuehe Ge, Zhizhang David Chen, Zhimeng Xu, Hai Zhang

**Affiliations:** 1 College of Information Science and Engineering, Huaqiao University, Xiamen, 361001 China; 2 College of Physics and Information Engineering, Fuzhou University, Fuzhou, 350108 Fujian Province China; 3 Department of Electrical and Computer Engineering, Dalhousie University, Halifax, Canada

**Keywords:** Optics and photonics, Physics

## Abstract

Optical metasurfaces are researched more and more intensively for the possible realization of lightweight and compact optical devices with novel functionalities. In this paper, a new beam-steering system based on double metasurface lenses (metalenses) is proposed and developed. The proposed system is lightweight, small volume, low cost, and easy to integrate. The exact close-form forward and numerical inverse solutions are derived respectively using the generalized Snell’s law of refraction. Given the orientations of the double metalenses, the pointing position can be accurately determined. If the desired pointing position is given, the required metalenses’ orientations can be obtained by applied global optimization algorithms to solve nonlinear equations related to the inverse problem. The relationships of the scan region and blind zone with the system parameters are derived. The method to eliminate the blind zone is given. Comparison with double Risley-prism systems is also conducted. This work provides a new approach to control light beams.

## Introduction

Steering light beams is very important and has found many applications in various optical systems, such as laser scanners, laser communications, laser radar, and other optical systems^1–3^. The common solutions to control light beams include using arrangements of galvo mirrors or pairs of rotatable Risley prisms. Risley prism systems (RPSs) are simple and low cost while particularly effective in continuously scanning the light beams; they have attracted much attention from researchers^1–16^ in the past several decades. Advantages of RPSs include compact size, stability, high precision and resolution, high reliability, etc. Different mathematical models on the forward and inverse formulas^4–16^ with different prism configurations have been implemented to provide scan patterns for the control of light beams. All these conventional methods are based on the classic refraction theorem^17^, namely Snell’s law.

Metamaterials, artificial materials capable of providing extraordinary electromagnetic responses not found in nature, have been the hot research topic^18,19^ in the past two decades. Especially in the past decade, considerable efforts^20–23^ have been devoted to the study of metasurfaces. Metasurfaces are two-dimensional (2D) metamaterials that feature subwavelength thickness, low loss, and easy fabrication, promising potential new applications. For the three-dimensional (3D) metamaterials, electrically, we are concerned about effective permittivity, permeability, and refractive index. For metasurfaces, we pay more attention to interface reflection and transmission, including their amplitude, phase, and polarization states. Abrupt phase changes can be introduced on ultrathin metasurfaces, leading to anomalous reflections and refractions that can be explained by generalized laws of reflection and refraction or called generalized Snell’s law^20^. They provide an alternative and efficient way to realize intriguing electromagnetic phenomena and devices, such as photonic spin Hall effect^24^, planar holograms^25^, polarization converters^21,26^, anomalous beam generators^20,27^, focusing lenses^28–32^. The planar gradient metasurfaces, based on the principle of rotatable Risley prisms, have been exploited for the design of low-profile scanning antennas operating in the microwave and millimeter-wave range^33–41^. Optical metasurfaces based on metallic nanostructures^42^, dielectric scatters^43^, and 2D materials such as graphene^44^ are also implemented to modulate the light beams. The new optical lens built on the metasurface platform, namely metalens, have become recent research hotspots for potential multifunctional applications.

In this paper, we develop a light beam scanner using two metasurface lenses (metalens). We apply gradient phases to metalens to redirect the impinging light beams. We use the generalized law of refraction to derive an accurate analytical relation between the pointing position and the orientations of the double metalens. We derive the inverse solution that allows finding the desired metalens’ orientations at any given pointing position.

The paper is organized as follows: Section 2 derives the exact analytic forward solution of the double-metalens scanner system. It also discusses the scanning accuracy, the scanning range, and the method to eliminate the scan blind zone. Section 3 develops an exact inverse solution. Section 4 concludes.

## The proposed metalens scanner

### Analysis

**Figure 1:**
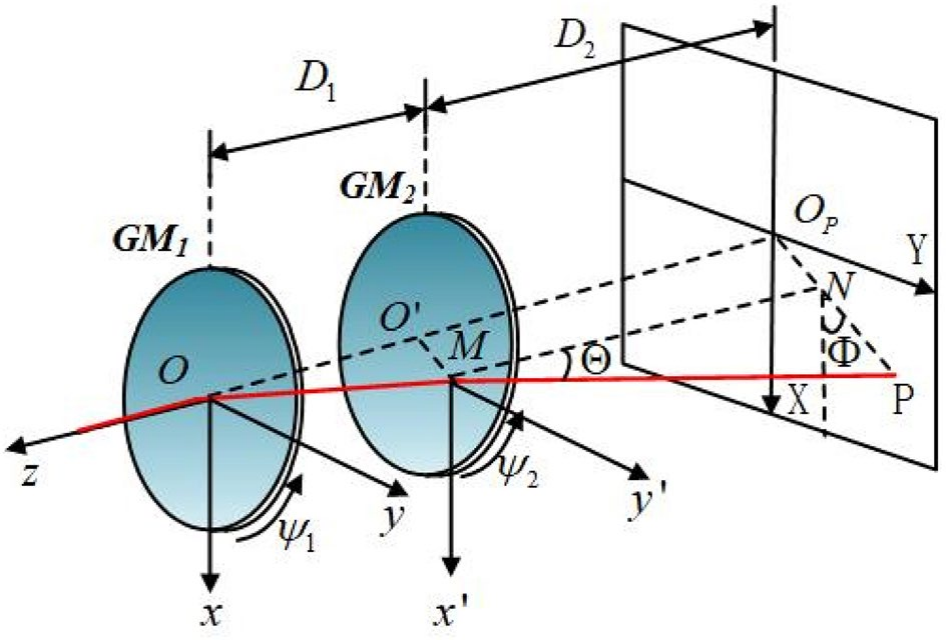
Schematic diagram of the proposed beam-steering double-metalens system.

The schematic of the proposed double-metalens scanner system under the Cartesian coordinate system is shown in Fig. 1. It consists of two metalenses (${GM}_{1}$ and ${GM}_{2}$) arranged along the z-axis, each of which has a planar structure, a subwavelength thickness, and a pre-determined phase gradient (${G}_{1}$ or ${G}_{2}$). The two metalenses can rotate around the z-axis independently. The parameters ${\psi }_{1}$ and ${\psi }_{2}$ represent the counterclockwise rotation angles of ${GM}_{1}$ and ${GM}_{2}$, respectively, with respect to the z-axis. The distances between the two metalenses and the second metalens and the receiving screen are D1 and D2, respectively. The incident light beam is supposed to be along the –z axis, passes Center O of ${GM}_{1}$ and Point M of ${GM}_{2}$, respectively, and finally arrives at Point P on the receiving screen. The Point M on ${GM}_{2}$ can be positioned with the longitudinal and azimuthal angles (θ, φ) with respect to Center O of ${GM}_{1}$. The position of Point P is determined by the coordinates (X, Y) on the receiving screen and the longitudinal and azimuthal angles (Θ, Φ) with respect to Point M of ${GM}_{2}$, respectively.

**Figure 2:**
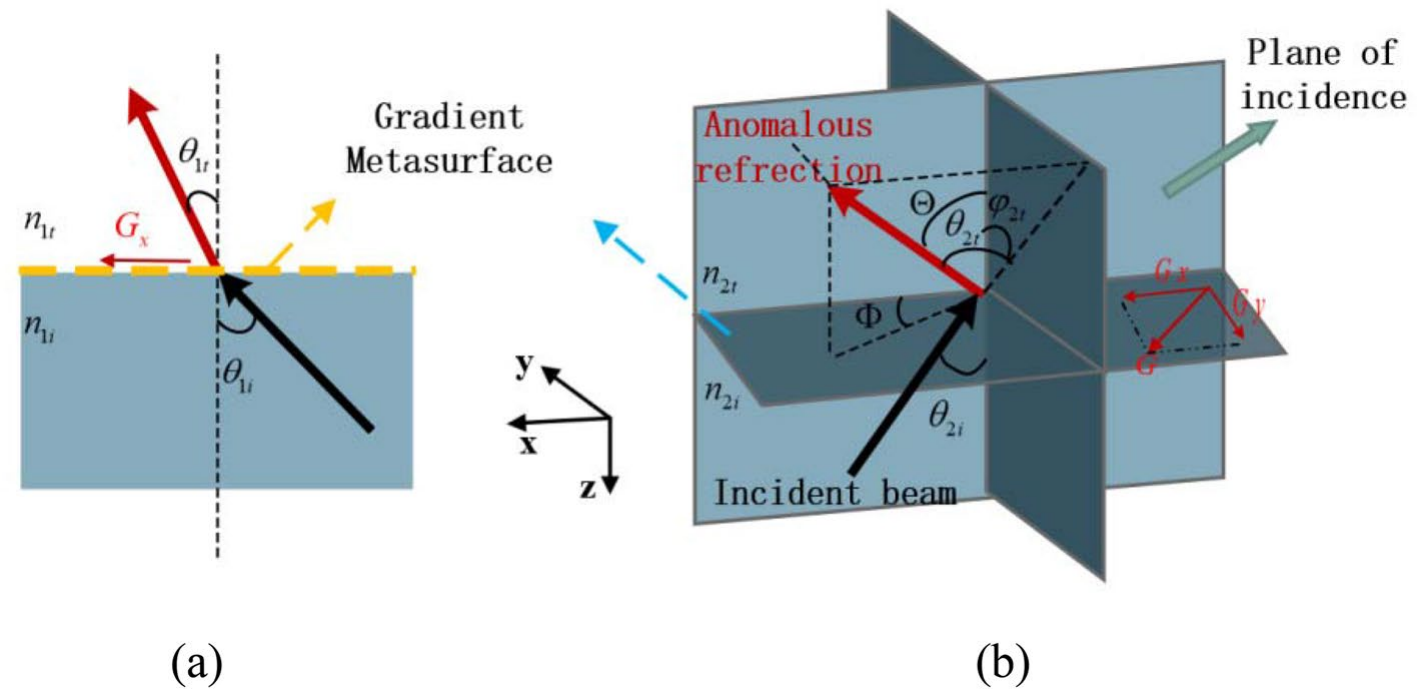
(**a**) 2D and (**b**) 3D generalized Snell’s law of refraction.

The relationship between the beam direction and the metalenses’ orientations is established based on the generalized Snell’s law^20^. In the initial state, the phase gradients ${G}_{1}$ and ${G}_{2}$ are along the x-axis, namely ${\psi }_{1}={\psi }_{2}=0$. The forward solution is to find out the beam pointing direction or the (X, Y) on the receiving screen with a set of given metalenses’ orientations. Assume ${\psi }_{1}$ and ${\psi }_{2}$ is known, the exact formulas can be derived using the generalized Snell’s law^20^. First, let us calculate the beam direction of the first metalens ${GM}_{1}$. The schematic for the 2D generalized Snell’s law of refraction is depicted in Fig. 2a. Based on the law, we have

1 $$\begin{array}{l}{n}_{t}sin{\theta }_{t}-{n}_{i}sin{\theta }_{i}=\frac{{\lambda }_{0}}{2\pi }\frac{d\phi }{dx} ,\end{array}$$

where ${n}_{t}$ and ${n}_{i}$ are the refractive index of the relative media, ${\lambda }_{0}$ is the wavelength of the incident wave, and $\frac{d\phi }{dx}$ is the phase gradient ${G}_{1}$, along x axis at the initial time.

As the beam is incident normally on ${GM}_{1}$, the direction of the leaving or transmitted beam can be expressed as:

2 $$\begin{array}{l}\left\{\begin{array}{l}{\theta }_{1t}=arcsin\left[\left(\frac{{G}_{1}}{{k}_{0}}+{n}_{i}sin{\theta }_{1i}\right)\frac{1}{{n}_{t}}\right]\\ {\varphi }_{1t}={\psi }_{1}\end{array}\right. ,\end{array}$$

where ${k}_{0}=\frac{2\pi }{{\lambda }_{0}}$ is the wavenumber. The metalenses are extraordinarily thin, and the media are air (or ${n}_{i}=1$ and ${n}_{t}=1$). The pointing direction (θ, φ) of the beam leaving ${GM}_{1}$ can be found as:

3 $$\begin{array}{l}\left\{\begin{array}{l}{\theta =\theta }_{1t}=arcsin\left(\frac{{G}_{1}}{{k}_{0}}\right)\\ \varphi ={\varphi }_{1t}={\psi }_{1}\end{array}\right. .\end{array}$$

Next, we derive the beam direction (Θ, Φ) of ${GM}_{2}$. From (3), the coordinates of the intersection point M on the second metalens is $({D}_{1}\mathrm{tan}{\theta }_{1t}\mathrm{cos}{\varphi }_{1t}, {D}_{1}\mathrm{tan}{\theta }_{1t}\mathrm{sin}{\varphi }_{1t}, -{D}_{1})$.

For the convenience of the derivation, a new coordinate system is set up. It has the origin at Point M and is rotated by an angle ${\psi }_{1}$ along the z-axis of the original coordinate system. The relative rotation angle of ${GM}_{2}$ in the new system is ${\psi }_{2}-{\psi }_{1}$ because ${GM}_{2}$ rotates by an angle of ${\psi }_{2}$ with respect to the original system. The rotation matrix on the new coordinates, denoted by $({x}^{\mathrm{^{\prime}}}, {y}^{\mathrm{^{\prime}}}, {z}^{\mathrm{^{\prime}}})$, is then given by:

4 $$\begin{array}{l}\left[\begin{array}{l}{x}^{\mathrm{^{\prime}}}\\ {y}^{\mathrm{^{\prime}}}\\ {z}^{\mathrm{^{\prime}}}\end{array}\right]=\left[\begin{array}{ccc}cos\left({\psi }_{2}-{\psi }_{1}\right)& -sin\left({\psi }_{2}-{\psi }_{1}\right)& 0\\ sin\left({\psi }_{2}-{\psi }_{1}\right)& cos\left({\psi }_{2}-{\psi }_{1}\right)& 0\\ 0& 0& 1\end{array}\right]\times \left[\begin{array}{l}x\\ y\\ z-{D}_{1}\end{array}\right].\end{array}$$

With the phase gradient ${G}_{2}$ initially along the x-axis on ${GM}_{2}$, corresponding to ${\overrightarrow{G}}_{2}={({G}_{2}, 0, 0)}^{T}$, the rotated phase gradient vector is given by

5 $$\begin{array}{l}{\overrightarrow{G}}_{2}^{\mathrm{^{\prime}}}=\left({G}_{x}, {G}_{y},{G}_{z}\right)=\left[{G}_{2}cos\left({\psi }_{2}-{\psi }_{1}\right),{G}_{2}sin\left({\psi }_{2}-{\psi }_{1}\right),0\right].\end{array}$$

With the 3D generalized Snell’s law, whose schematic is shown in Fig. 2b, the following formula is obtained

6 $$\begin{array}{l}\left\{\begin{array}{l}cos{\theta }_{2t}sin{\varphi }_{2t}=\frac{{G}_{y}}{{{n}_{2t}k}_{0}}\\ {n}_{2t}sin{\theta }_{2t}-{n}_{2i}sin{\theta }_{2i}=\frac{{G}_{x}}{{k}_{0}}\end{array}\right..\end{array}$$

With ${n}_{2i}={n}_{2t}=1$ and ${\theta }_{2i}=\theta$, $\left({\theta }_{2\mathrm{t}}, {\varphi }_{2\mathrm{t}}\right)$ can be derived from (6)

7 $$\begin{array}{l}\left\{\begin{array}{l}{\theta }_{2t}=arcsin\left[\frac{{G}_{1}}{{k}_{0}}+\frac{{G}_{2}cos\left({\psi }_{2}-{\psi }_{1}\right)}{{k}_{0}}\right]\\ {\varphi }_{2t}=arcsin\left[\frac{{G}_{2}sin\left({\psi }_{2}-{\psi }_{1}\right)}{{k}_{0}cos{\theta }_{2t}}\right]\end{array}\right. .\end{array}$$

Then by transforming (${\theta }_{2t}, {\varphi }_{2t}$) into the spherical coordinate (θ_2_, φ_2_) on the new coordinate system, we have

8 $$\begin{array}{l}\left\{\begin{array}{l}{\theta }_{2}=arccos\left(cos{\theta }_{2t}cos{\varphi }_{2t}\right)\\ {\varphi }_{2}=arcsin\left(\frac{cos{\theta }_{2t}sin{\varphi }_{2t}}{sin{\theta }_{2}}\right)\end{array}\right. .\end{array}$$

By rotating the new coordinate system back to its original, we have:

9 $$\begin{array}{l}\left(\Theta ,\Phi \right)=\left({\theta }_{2},{\varphi }_{2}+{\psi }_{1}\right).\end{array}$$

The coordinate $(\mathrm{X},\mathrm{Y})$ of Point P on the receiving screen is obtained by

10 $$\begin{array}{l}\left\{\begin{array}{l}X={D}_{1}tan\theta cos\varphi +{D}_{2}tan\Theta cos\Phi \\ Y={D}_{1}tan\theta sin\varphi +{D}_{2}tan\Theta sin\Phi \end{array}\right. .\end{array}$$

### The scan blind zone

**Figure 3:**
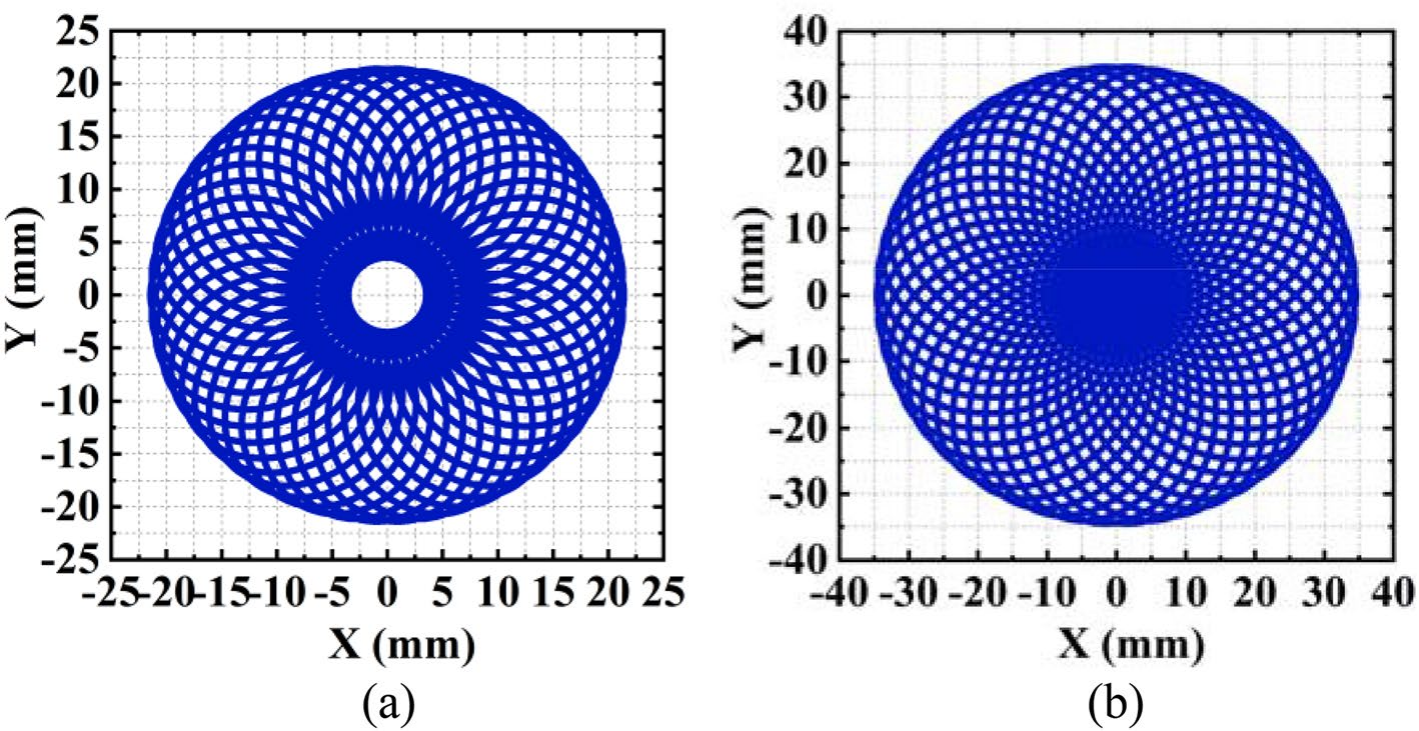
The beam scan regions (**a**) with the blind zone and (**b**) with the beam blind zone eliminated.

A scan blind zone occurs when the phase gradients of the two metalenses are identical, leading to the target lost within the scan region. Here an example is given to illustrate and discuss the problem. With ${G}_{1}$=${G}_{2}$=4188.8 rad/mm, ${D}_{1}=10\, \text{mm}$, ${D}_{2}=20\, \text{mm}$, and ${\lambda }_{0}=500 \,\text{nm}$ (corresponding to ${k}_{0}=0.0126\, \text{nm}^{-1}$), the scan region on the receiving screen is calculated and plotted in Fig. 3a. There is a circular blind zone in the center of the circular scan region. Based on the forward solution above, the blind zone is determined by the phase gradient and occurs when the gradients of ${\mathrm{GM}}_{1}$ and ${\mathrm{GM}}_{2}$ are identical. Based on the geometrical relationship between the leaving beam of ${\mathrm{GM}}_{1}$ and the intersection point $\mathrm{M}$, the radius of the blind zone, |O’M|, is:

11 $$\begin{array}{l}{|O}^{\mathrm{^{\prime}}}M|={D}_{1}\mathrm{tan}\left({\theta }_{1t}\right)={D}_{1}\mathrm{tan}\left(\theta \right).\end{array}$$

Since the altitude angle θ increases with the phase gradient G1, the blind zone will increase with ${D}_{1}$ and ${G}_{1}$.

Increasing the phase gradient ${G}_{2}$ of ${\mathrm{GM}}_{2}$ is a solution to decrease or eliminate the scan blind zone. From (7) and (8), when $\left|{\psi }_{2}-{\psi }_{1}\right|=180^\circ$, we have:

12 $$\begin{array}{l}{\theta }_{2}={\theta }_{2\mathrm{t}}=arcsin\left[\frac{{G}_{1}}{{k}_{0}}-\frac{{G}_{2}}{{k}_{0}}\right].\end{array}$$

With the geometrical relationship between the emerging beam of ${\mathrm{GM}}_{2}$ and the scan point $\mathrm{P}$ on the receiving screen, we get:

13 $$\begin{array}{l}\left|NP\right|={D}_{2}\mathrm{tan}\left(\theta \right).\end{array}$$

Combining (11), (12), and (13), we have the increment of ${G}_{2}$:

14 $$\begin{array}{l}\Delta G={\left|{G}_{2}-{G}_{1}\right|=k}_{0}\mathrm{sin}\left(\mathrm{atan}\left(\frac{\left[O\mathrm{^{\prime}}M\right]}{{D}_{2}}\right)\right).\end{array}$$

Figure 3b plots the scan region without a blind zone, where the increment of ${G}_{2}$ is 2187.5 rad/mm, corresponding to an enlarged phase gradient ${\mathrm{G}}_{2}^{\mathrm{^{\prime}}}=$ 6376.3 rad/mm.

### The scan region

In Fig. 3, not only the blind zone is eliminated, but also the scan region enlarged when the phase gradient ${G}_{2}$ increases. Therefore, it is necessary to discuss the impact of phase gradients on the scan region. The maximum X and Y of the scan region on the receiving screen can be obtained when $\left|{\psi }_{2}-{\psi }_{1}\right|=0^\circ$. From (10), we have:

15 $$\begin{array}{l}{X}_{max}={Y}_{max}={D}_{1}\mathrm{tan}\left[\mathrm{arcsin}\left(\frac{{G}_{1}}{{k}_{0}}\right)\right]+{D}_{2}\mathrm{tan}\left[\mathrm{arcsin}\left(\frac{{G}_{1}}{{k}_{0}}+\frac{{G}_{2}}{{k}_{0}}\right)\right].\end{array}$$

**Figure 4:**
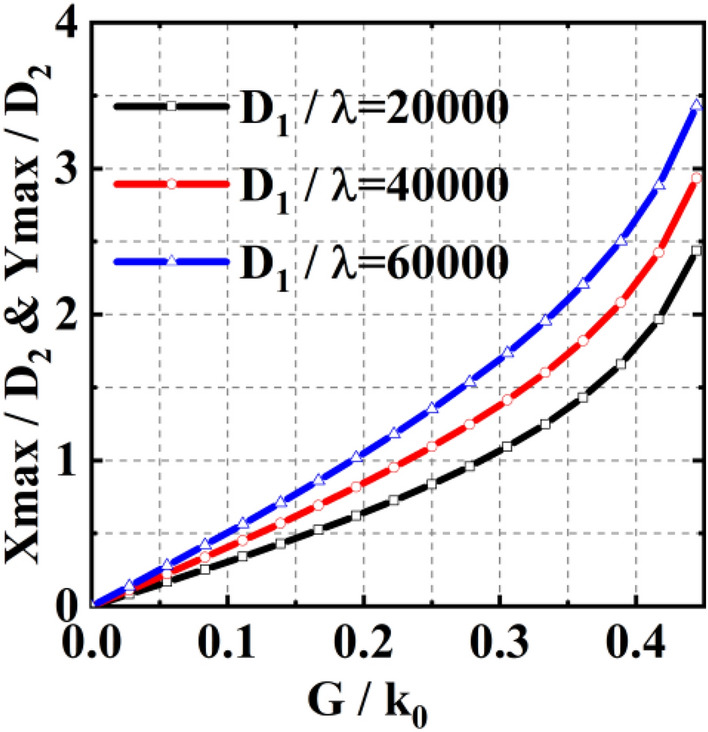
Influence of ${D}_{1}$, ${D}_{2}$, and $G/{k}_{0}$ on the beam scan region defined by (${X}_{max}$, ${Y}_{max}$).

Next, we show how the scan region varies with the parameters of the double-metalens system. For the convenience of the analysis, the phase gradients of ${\mathrm{GM}}_{1}$ and ${\mathrm{GM}}_{2}$ are set to be identical ($G={G}_{1}={G}_{2}$). Figure 4 illustrates the relations among (Xmax, Ymax), ${D}_{1}/{\lambda }_{0}$ , ${D}_{2}$, and $G/{k}_{0}$. It is obvious that the scan region will increase with ${D}_{1}/{\lambda }_{0}$ , ${D}_{2}$, and $G/{k}_{0}$.

### Impacts of the distance between metalenses

The distance between metalenses could not be too large. If the two metalenses are placed too far away from each other, the emerging beam from ${GM}_{1}$ may propagate beyond the aperture of ${GM}_{2}$. For example, if two identical metalenses are taken with aperture $D=200\mathrm{ mm}$ and $\frac{{G}_{1}}{{k}_{0}}=0.33$, the elevation angle ${\theta }_{1t}$ is $19.47^\circ$ and the maximum distance can be calculated below:

16 $$\begin{array}{l}{D}_{1max}=\frac{D/2}{\mathrm{tan}\left({\theta }_{1t}\right)}=282.8 \text{mm}.\end{array}$$

In practice, a smaller distance is preferred to reduce the scan blind zone^45^. However, if the distance between ${GM}_{1}$ and ${GM}_{2}$ is too close, mutual coupling between metalenses may become unacceptly large. Hence the compromised distance value should be taken in practice, based on the metasurfaces utilized. In addition, the minimum distance is dependent on the specific characteristics of the metelenses, while the maximum distance on the wavelength of the incident beam, the size of the lens aperture, and the phase gradients of the metalenses.

**Figure 5:**
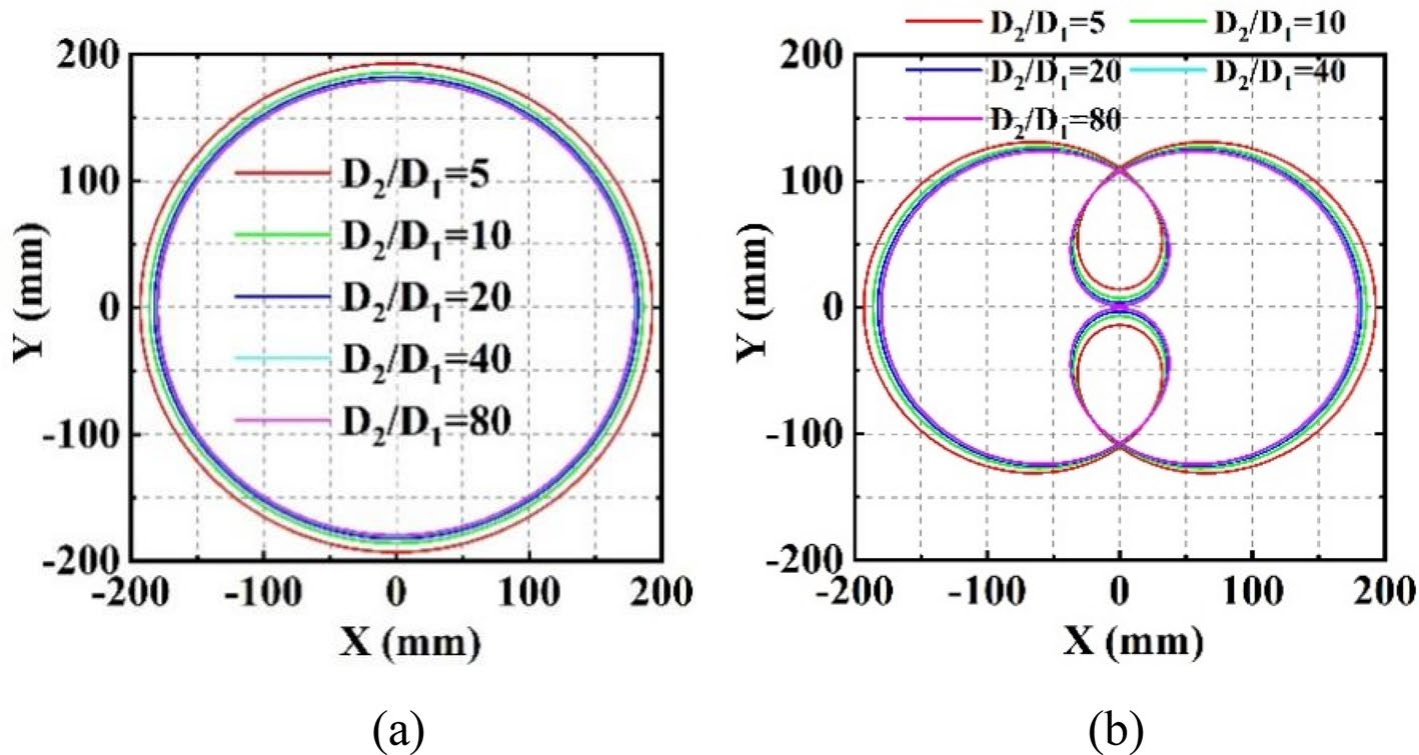
Beam trajectories with different ${D}_{2}/{D}_{1}$ ratios (**a**) ${\omega }_{1}={\omega }_{2}$ (**b**) ${\omega }_{1}={3\omega }_{2}$, where ${\omega }_{1}$ and ${\omega }_{2}$ are the rotationally angular speed of the two metalens^12,45^.

The distance ${D}_{1}$ also impacts the beam trajectories^12,45^ because it affects the scan region. Figure 5 illustrates the differences in trajectories when ${D}_{2}$ is constant. It is seen that the impact of ${D}_{1}$ can be ignored when ${D}_{2}/{D}_{1}$ ratio is larger than 40.

### Comparison with the paraxial approximation solution of risley-prism systems

In the conventional double Risley-prism systems, the first-order paraxial method^8,45^ is a typical solution for RPSs with thin prisms that have small deviation angles. However, since the first-order approximation is applied, it only works when the approximation is good enough. Next, comparisons with the first-order method^8,45^ with RPSs are conducted to demonstrate the accuracy of the proposed method. The mathematical expressions of the first-order method expressions are:

17 $$\begin{array}{l}\left\{\begin{array}{l}\Theta =\sqrt{{\delta }_{1}^{2}+{\delta }_{2}^{2}+2{\delta }_{1}{\delta }_{2}\mathrm{cos}\left({\psi }_{1}-{\psi }_{2}\right)}\\ \Phi =\mathrm{arctan}\left(\frac{{\delta }_{1}\mathrm{sin}\left({\psi }_{1}\right)+{\delta }_{2}\mathrm{sin}\left({\psi }_{2}\right)}{{\delta }_{1}\mathrm{cos}\left({\psi }_{1}\right)+{\delta }_{2}\mathrm{cos}\left({\psi }_{2}\right)}\right)\end{array}\right.,\end{array}$$

where ${\delta }_{1}$ and ${\delta }_{2}$ denote the deviation angles by the two prisms, respectively. The relationship between the deviation angle and the phase gradient with the proposed double-metalens scanner system can be obtained from (3):

18 $$\begin{array}{l}\delta =\mathrm{arcsin}\left(\frac{G}{{k}_{0}}\right).\end{array}$$

In the comparison, the double-metalens scanner system with two identical metalenses is considered. Let $\frac{{G}_{1}}{{k}_{0}}$ and $\frac{{G}_{2}}{{k}_{0}}$ be 0.25 and 0.33, respectively, which corresponds to the opening angles of 28.96° and 38.94° of the prisms, respectively, the comparisons are made between the first-order method and the proposed analytic solution. The beam trajectory is acquired by keeping ${\psi }_{1}=90^\circ$ and varying ${\psi }_{2}$ from $0^\circ$ to $360^\circ$.

**Figure 6:**
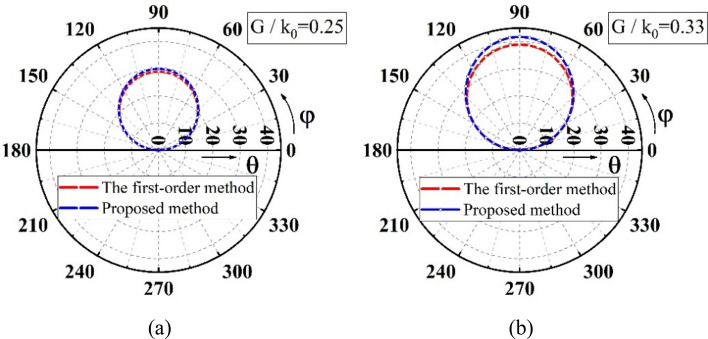
Comparisons for the beam trajectory when ${\psi }_{1}=90^\circ$ and ${\psi }_{2}$ varies from $0^\circ$ to $360^\circ$. (**a**) G/${k}_{0}=0.25$; (**b**) G/${k}_{0}=0.33$.

Figure 6 shows the two comparison results. When the phase gradient is small, corresponding to the small opening angle of the prism, the two results are relatively close (see Fig. 6a). When the phase gradient or the opening angle increases, the differences between the two results increase (see Fig. 6b). The reason is that increasing the opening angle of the prism will introduce a larger error due to the first-order approximation^8,45^ while increasing the phase gradient will not change the accuracy of the proposed analytical solution. An increase of the phase gradient of the metalens or the opening angle of the prism in the two systems leads to the increase of the scanning region. Therefore, the proposed metalens system always gives accurate solutions for any pre-determined scanning region without changing the volume of the system.

## Inverse solution and target tracking performance for double-metalens systems

### Inverse solution

The inverse solution is to find out the orientation angles of the metalenses for a given pointing position on the receiving screen. It is especially significant in practical applications such as target tracking. Following the exact derivation of the forward solution of the new double-metalens system, an exact inverse solution is found as follows.

Equation (8) can be rewritten in the following form when substituting (9) into (8):

19 $$\begin{array}{l}\left\{\begin{array}{l}cos\theta =cos{\theta }_{2t}cos{\varphi }_{2t}\\ sin\theta =\frac{cos{\theta }_{2t}sin{\varphi }_{2t}}{sin\left(\varphi -{\psi }_{1}\right)}\end{array}\right..\end{array}$$

Then (19) can be further derived as:

20 $$\begin{array}{l}tan{\theta }_{2t}=tan\theta sin\left(\varphi -{\psi }_{1}\right),\end{array}$$

21 $$\begin{array}{l}{cos}^{2}{\theta }_{2t}=1-{sin}^{2}\theta {cos}^{2}\left(\varphi -{\psi }_{1}\right).\end{array}$$

Let $\mathrm{A}=\frac{{G}_{1}}{{k}_{0}}$ and $\mathrm{B}=\frac{{G}_{2}}{{k}_{0}}$, the new expressions of (7) are:

22 $$\begin{array}{l}sin{\theta }_{2t}=A+Bcos\left({\psi }_{2}-{\psi }_{1}\right),\end{array}$$

23 $$\begin{array}{l}{sin\varphi }_{2t}{cos\theta }_{2t}=Bsin\left({\psi }_{2}-{\psi }_{1}\right).\end{array}$$

Substituting (21) into (22), we get:

24 $$\begin{array}{l}sin\theta cos\left(\varphi -{\psi }_{1}\right)=A+Bcos\left({\psi }_{2}-{\psi }_{1}\right).\end{array}$$

Solving (8), (9), and (23) simultaneously, we have:

25 $$\begin{array}{l}sin\theta sin\left(\varphi -{\psi }_{1}\right)=Bsin\left({\psi }_{2}-{\psi }_{1}\right).\end{array}$$

The orientations ${\psi }_{1}$ and ${\psi }_{2}$ can be obtained by solving (10), (24), and (25) through optimization algorithms, for example, genetic algorithm, particle swarm optimization algorithm, differential evolution algorithm, etc. However, they usually converge slowly. For example, with a random initial value, the differential evolution algorithm will take thousands of iterations to converge and find a correct solution for the inverse problem, whereas the other two algorithms need more iterations and times. In this work, we used the MATLAB function vpasolve to get the optimized solutions, which is more efficient for the solution.

### Target tracking performance

An asteroid target trajectory is applied to validate the performance of the target tracking using the proposed inverse solution; the expression of the asteroid is:

26 $$\begin{array}{l}\left\{\begin{array}{l}X=100{cos}^{3}\left(\theta \right)\\ Y=100{sin}^{3}\left(\theta \right)\end{array}, 0\le \theta \le 2\pi \right..\end{array}$$

The MATLAB function vpasolve is used for the solution. This function can be used to find all the numerical solutions of algebraic equations with a random starting point. The fitness function or the algebraic equation finding the target trajectory is the sum of the absolute value of (10), (24), and (25).

**Figure 7:**
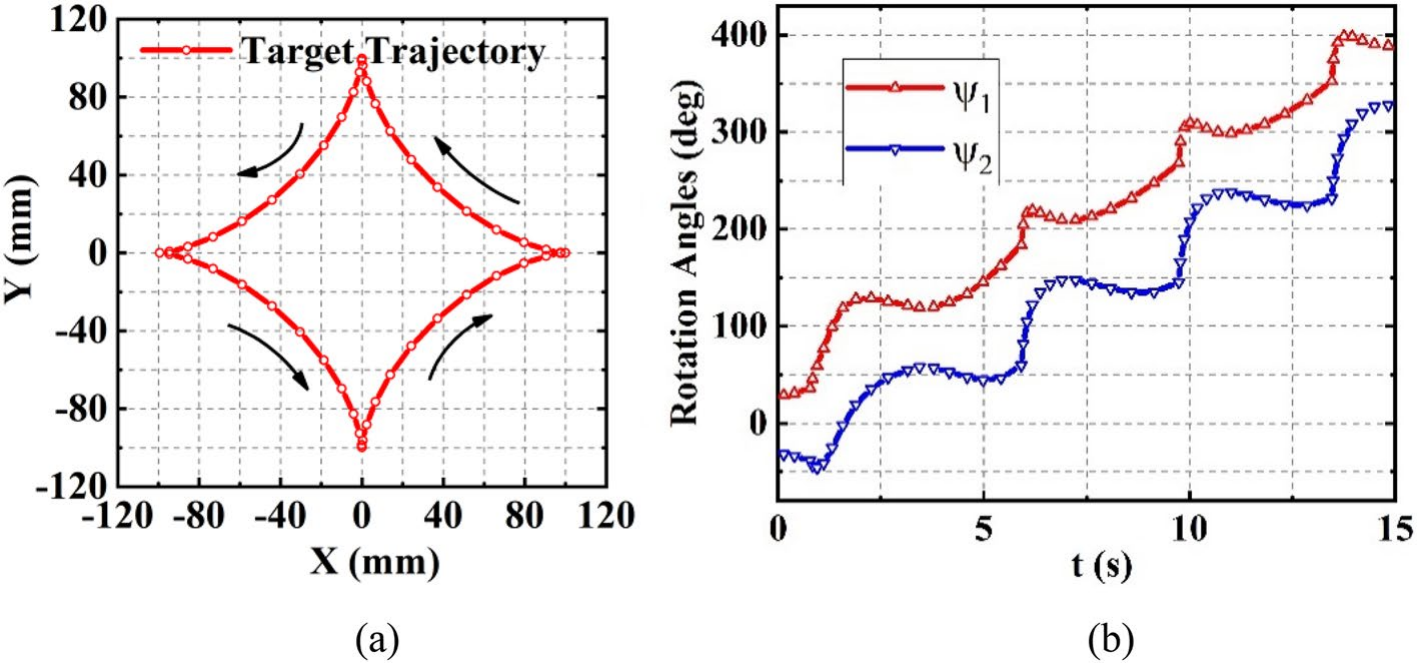
(**a**) The given asteroid target trajectory, (**b**) the inverse solutions to the orientations of ${\mathbf{G}\mathbf{M}}_{1}$ and ${\mathbf{G}\mathbf{M}}_{2}$.

With $A=B=0.33$, ${\mathrm{D}}_{1}=20\,\text{mm}$, and ${\mathrm{D}}_{2}=200\,\text{mm}$, the trajectory and the inverse solutions to ${\psi }_{1}$ and ${\psi }_{2}$ are plotted in Fig. 7a,b, respectively. Substituting the orientations obtained from the inverse solution into the analytic forward solution, we found that the errors are less than 1 μm.

## Discussion and conclusions

The optical metalens is currently an important under-explored topic. There are two kinds of metalenses: metallic plasmonic one and all-dielectric one. The latter is free from ohmic losses, leading to a much higher efficiency of operation. The beam-steering systems-based metalenses are lightweight, compact, and easy to be integrated because the volumes of metalenses do not vary much with the change of phase gradients.

The exact forward and inverse solutions for beam-steering systems with double metalenses are successfully derived based on the generalized Snell’s law of refraction in this paper, where the forward one is in close form while the inverse one should be obtained by the numerical method. The relationships among the scan region, the blind zone, the beam trajectory, and system parameters are derived or discussed. The approach to eliminating the scan blind zone is presented and numerically demonstrated. Comparison of the new system with the conventional Risley-prism systems is carried out, demonstrating that the proposed method can give effective and accurate results.

There are some limitations with the current double-metalens beam-steering systems. Due to the dispersions of metalenses and the refraction, they are more applicable to monochromatic light beams. The optimization algorithms to solve the inverse solution converge slowly. More efficient methods are needed for practical applications.

Although the Risley-prism beam-steerable approach has inspired the mechanically beam-steerable antennas^15,16,33–41^ with gradient metasurface in the microwave and millimeter-wave range, the solutions are only applicable to radiation beams with the infinity. The blind zone concept does not exist in antenna works^15,16,33–41^. In the optical range, the distances between the light source and the beam trajectories are finite. One has to consider the distance between the two metasurfaces and that between the second metasurface (**GM**_**2**_ in Fig. 1) and the target trajectory screen. The forward and inverse solutions^15,16,33–41^ in the microwave and millimeter-wave range cannot be directly applied to find the optical beam trajectory.
